# Maternal antecedents of adiposity and studying the transgenerational role of hyperglycemia and insulin (MAASTHI): a prospective cohort study: *Protocol of birth cohort at Bangalore, India*

**DOI:** 10.1186/s12884-016-1088-4

**Published:** 2016-10-14

**Authors:** Giridhara R. Babu, GVS Murthy, R. Deepa, Yamuna, Prafulla, H. Kiran Kumar, Maithili Karthik, Keerti Deshpande, Sara E. Benjamin Neelon, D. Prabhakaran, Anura Kurpad, Sanjay Kinra

**Affiliations:** 1 Wellcome Trust-DBT India alliance Intermediate Research Fellow in Public Health, Additional Professor, Public Health Foundation of India (PHFI), IIPH-H, Bangalore campus, SIHFW premises, Beside leprosy hospital, 1st cross, Magadi road, Bangalore, 560023 India; 2 Indian Institute of Public Health-Hyderabad, Plot # 1, A.N.V. Arcade, Amar Co-op Society, Kavuri Hills, Madhapur, Hyderabad, 500033 India; 3 ICEH, London School of Hygiene & Tropical Medicine, 3rd Floor, South Courtyard, Keppel Street, London, WC1E 7HT UK; 4 Research team of MAASTHI, Public Health Foundation of India, IIPH-H, Bangalore campus, SIHFW premises, Beside leprosy hospital, 1st cross, Magadi road, Bangalore, 560023 India; 5 Johns Hopkins Bloomberg School of Public Health, 624 N Broadway, Hampton House 755, Baltimore, MD 21205 USA; 6 Centre for Diet and Activity Research, MRC Epidemiology, University of Cambridge, Cambridge, UK; 7 Centre for Control of Chronic Conditions (CCCC, Public Health Foundation of India (PHFI), New Delhi, India; 8 Centre for Chronic Disease Control (CCDC), New Delhi, India; 9 Department of Epidemiology, London School of Hygiene and Tropical Medicine, London, WC1E 7HT UK; 10 Emory Rollins School of Public Health, 1518 Clifton Rd, Atlanta, GA 30322 USA; 11 Nutrition Division, St John’s Research Institute, Bangalore, India; 12 Nutrition Society of India, National Institute of Nutrition Campus, Hyderabad, 500 007 India; 13 IAEA Collaborating Centre for Stable Isotope Technologies in Nutrition, St John’s Research Institute, Bangalore, India; 14 International Nutrition Foundation Protein Advisory Group and Chair, Expert Committee on Obesity, ICMR, New Delhi, India; 15 Reader in Clinical Epidemiology & Honorary Consultant in Paediatric Obesity - London School of Hygiene & Tropical Medicine & University College London Hospital, London, UK

**Keywords:** Gestational diabetes, Hyperglycemia, Obesity, Birth cohort, Protocol, Lifecourse epidemiology

## Abstract

**Background:**

India is experiencing an epidemic of obesity-hyperglycaemia, which coincides with child bearing age for women. The epidemic can be sustained and augmented through transgenerational transmission of adiposity and glucose intolerance in women. This presents an opportunity for exploring a clear strategy for the control of this epidemic in India. We conducted a study between November 2013 and May 2015 to inform the design of a large pregnancy cohort study. Based on the findings of this pilot, we developed the protocol for the proposed birth cohort of 5000 women, the recruitment for which will start in April 2016. The protocol of the study documents the processes which aim at advancing the available knowledge, linking several steps in the evolution of obesity led hyperglycemia.

**Methods:**

Maternal Antecedents of Adiposity and Studying the Transgenerational role of Hyperglycemia and Insulin (MAASTHI) is a cohort study in the public health facilities in Bangalore, India. The objective of MAASTHI is to prospectively assess the effects of glucose levels in pregnancy on the risk of adverse infant outcomes, especially in predicting the possible risk markers of later chronic diseases. The primary objective of the proposed study is to investigate the effect of glucose levels in pregnancy on skinfold thickness (adiposity) in infancy as a marker of future obesity and diabetes in offspring. The secondary objective is to assess the association between psychosocial environment of mothers and adverse neonatal outcomes including adiposity. The study aims to recruit 5000 pregnant women and follow them and their offspring for a period of 4 years. The institutional review board at The Indian Institute of Public Health (IIPH)-H, Bangalore, Public Health Foundation of India has approved the protocol. All participants are required to provide written informed consent.

**Discussion:**

The findings from this study may help to address important questions on screening and management of high blood sugar in pregnancy. It may provide critical information on the specific determinants driving the underweight-obesity-T2DM epidemic in India. The study can inform the policy regarding the potential impact of screening and management protocols in public healthcare facilities. The public health implications include prioritising issues of maternal glycemic control and weight management and better understanding of the lifecourse determinants in the development of T2DM.

**Electronic supplementary material:**

The online version of this article (doi:10.1186/s12884-016-1088-4) contains supplementary material, which is available to authorized users.

## Background

India is facing an epidemic of Type II Diabetes Mellitus (T2DM) with 69.2 million people with diabetes in 2015 [1]. This prevalence rate is projected to increase to 87 million in 2030. [2] T2DM affects Indians earlier than developed countries, [2–7] and yet there is limited research to elucidate the causal mechanisms. Part of this high burden may be explained by programming of T2DM and obesity ensuing in early life [8–12]. Hyperglycemia in pregnant women may alter the intrauterine environment, thereby increasing the risk of obesity in childhood and their future risk of diabetes.[2, 12–19] With an estimated 16.9 % of pregnant women affected [13], approximately 6 million women might be suffering from hyperglycemia in pregnancy in India [1, 20]. The severity and form of maternal hyperglycemia may have a distinct role in the development of childhood obesity and T2DM in adult life [19, 21–23]. It is important to recognize the specific deleterious outcomes which may manifest in children, including effects of maternal carbohydrate intolerance at all levels including near normal levels [16, 24–27]. Understanding this pathway could help address the underweight and gestational hyperglycemia led obesity epidemic in low- and middle-income countries (LMICs) like India.

Hyperglycemia and Adverse Pregnancy Outcome (HAPO) study confirmed the link between maternal glucose and neonatal adiposity and suggested that the relationship might be mediated by fetal insulin production. [27–30]. From the lifecourse perspective, the epistemology for the gestational hyperglycemia obesity led epidemic has been implied by three important hypotheses. The ‘fuel mediated teratogenesis’ hypothesis [27, 30] states that in-utero exposure to maternal hyperglycaemia can result in fetal hyperinsulinaemia [8, 27, 30–32]. The ‘thrifty phenotype’ hypothesis suggests that adaptive mechanisms due to child undernutrition may result in T2DM epidemic in LMICs [4]. The ‘thrifty genotype’ hypothesis infers that conservatory mechanisms at the level of the genotype can occur in populations that have undergone a sudden transition from undernutrition to overnutrition [9]. The ‘thrifty insulin hypothesis’ forms a part of this, which states that low birth weight and T2DM are two phenotypes of the same genotype and also predict insulin resistance [9]. The initial evidence for these hypotheses is provided by the Parthenon Birth cohort and similar studies [19, 33–35].

Urbanization-led changes in Indians with contributions from unique genotype and phenotype is likely contributing to the epidemic of obesity-hyperglycaemia. India is experiencing a rise in prevalence of adiposity and glucose intolerance at younger ages, which coincides with child bearing age for women [30]. The later stages of the epidemic could then be sustained and augmented by a feed forward mechanism, through transgenerational transmission of adiposity and glucose intolerance in women [36–38]. This presents an opportunity for exploring a clear strategy for the control of this epidemic in India through rigorous screening and management of adiposity and glucose intolerance in pregnancy.

Between November 2013 and May 2015, we conducted a pilot to inform the design of a large pregnancy cohort study that could reliably test some aspect of the above-mentioned hypotheses. The protocol of the initial study and the baseline results have already been published [39]. Based on the findings of this pilot, we developed the protocol for the proposed pregnancy cohort of 5000 women, the recruitment for which began in April 2016. The protocol of the study documents the processes which aim at advancing the available knowledge, linking several steps in the evolution of T2DM.

## Methods/design

### Aim and objectives

**Fig. 1:**
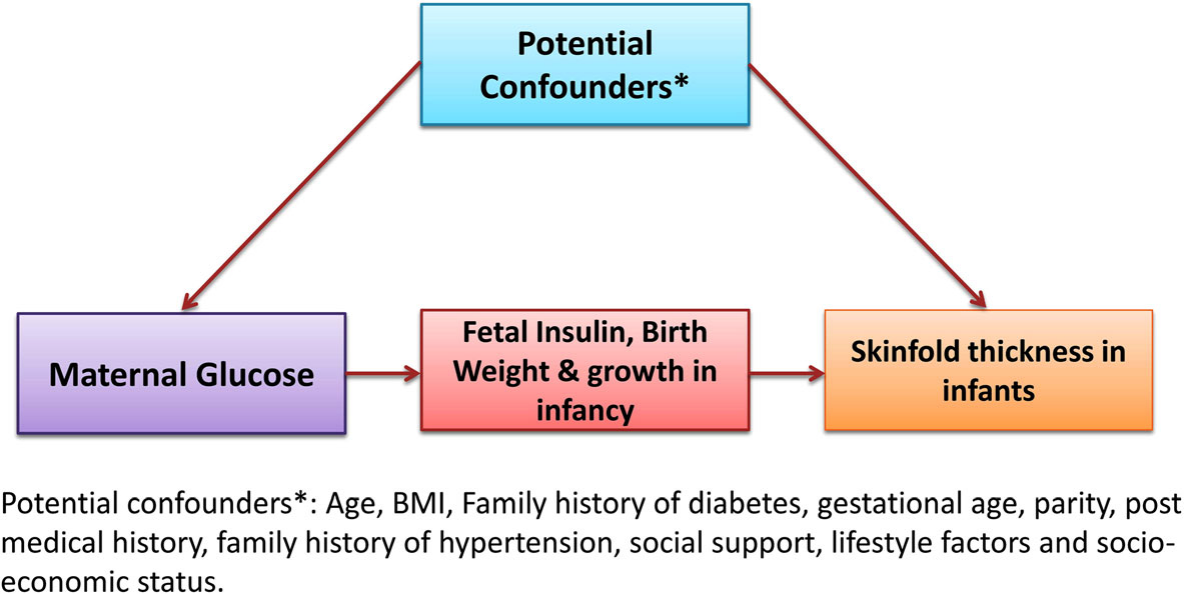
Conceptual Directional Acyclic Graph (DAG) depicting the hypothesis 1. Maternal glucose in pregnancy is associated with skinfold thickness (adiposity) of offspring at one year

The aim of this study is to prospectively assess the effects of glucose levels in pregnancy on the risk of adverse infant outcomes, especially in predicting the possible risk markers of later chronic diseases (Fig. 1). The primary objective of the proposed study is to investigate the effect of glucose levels in pregnancy on skinfold thickness (adiposity) in infancy as a marker of future obesity and diabetes in offspring. Because psychosocial environment could itself be an important independent predictor of adiposity and could confound its relationship with maternal hyperglycaemia, our secondary objective is to assess the association between psychosocial environment of mothers and adverse neonatal outcomes including adiposity. The specific exposure, outcome and potential confounders for these objectives are listed in Table 1.

**Table 1:** The exposure, outcome and potential confounders for the study objectives

Exposure	Outcome	Potential confounders
Maternal glucose level	Skinfold thickness in infants at one year	Age, body mass index (BMI), family history of diabetes, gestational age, parity, past medical history, family history of hypertension and socio-economic status
Psychosocial environment	Skinfold thickness in infants at one year	Maternal age, parity, BMI, weight-gain during pregnancy on fetal biometry measures, diet, gestational age, lifestyle factors, alcohol and tobacco use

### Hypotheses:

**Fig. 2:**
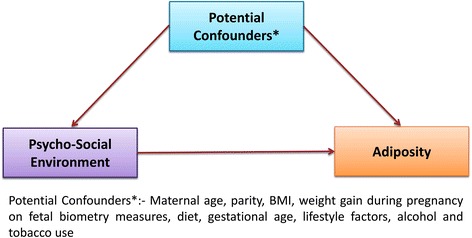
Conceptual Directional Acyclic Graph (DAG) depicting the hypothesis 2. Psychosocial environment measured by social support and distress is related to skinfold thickness of offspring at one year

1. Maternal glucose in pregnancy is associated with skinfold thickness (adiposity) of offspring at one year (Fig. 1)
2. Psychosocial environment measured by social support and distress is related to skinfold thickness of offspring at one year (Fig. 2)

For hypotheses 1, the exposure of interest is maternal blood glucose level. We are not examining gestational diabetes only, but are interested in exploring the effect of the continuous range of glucose as exposure from near normal ranges to hyperglycemia.

After the fasting blood sample is drawn, the pregnant woman will receive 75 g oral glucose load and a venous blood sample will be collected 2 hours later for estimating plasma glucose [40–42]. Evaluation of the samples will be done through the central laboratory so as to minimize the confounding effects of analytic variation. The primary outcome of our study is skinfold thickness in infants as a measure of adiposity. Skinfold thickness is the most accurate method of measuring body fat rather than Body Mass Index (BMI) as this will provide a better indication of individual fat and risk of obesity. Skinfold thickness measurement is a traditional and reliable measure of fat percentage. From research it’s found that greater weight gain during pregnancy was associated with higher child’s body mass index, sum of triceps and subscapular skinfold thickness [43, 44]. The triceps, biceps and subscapular skinfolds will be measured as described by Tanner/Whitehouse using a Holtain Calipers (Holtain, U.K.). Triceps skin folds will be measured over the posterior belly of triceps muscle of the left arm, halfway between the acromion and the olecranon, on a line passing upwards from the olecranon in the axis of the limb, with the arm extended. Subscapular skinfold will be measured immediately below the angle of the left scapula, in the natural cleavage line of the skin, with the arm held by the side of the body. In addition, we will also assess weight for length and waist girth (in centimeters) as co-primary outcomes. Waist girth is measured by placing the tape around the abdomen immediately above the umbilicus ensuring that it is horizontal and marked at the end of expiration. The ultrasound scan records of the respondents are reviewed and the estimated foetal weight, biparietal diameter (BPD), head circumference (HC), abdominal circumference (AC), femur length (FL) is recorded. Mother’s weight and skinfold thickness are measured during antenatal period. Based on the literature, we have identified the following potential confounders based on a priori knowledge: age, BMI, family history of diabetes, gestational age, parity, past medical history, family history of hypertension and socio-economic status.

For hypotheses 2, the exposure of interest is social support, measured by means of a validated tool (Questionnaire) administered during second trimester of pregnancy. Social support to the mother will be measured using a questionnaire developed at St. John’s Research Institute to evaluate a broad range of social support (i.e., emotional, instrumental, informational, and appraisal) [45]. The Edinburgh Postnatal Depression Scale is administered to the respondents during pregnancy, after delivery and when the infant is 14 week old. Those who score greater than 13 in the EPDS scale are administered Global Mental Health Assessment Tool (GMHAT) for further diagnosis and are referred to the psychiatrist at the hospital for counselling and treatment. The outcome of interest is infant adiposity. The potential confounders include maternal age, parity, BMI, weight gain during pregnancy on fetal biometry measures, [46] diet, gestational age, lifestyle factors, and alcohol and tobacco use [46, 47]. The mother’s 24 hour diet recall is recorded during pregnancy. The infant’s feeding mode whether it is breastfed, time of first breast feed and exclusive breastfeeding at birth, duration of breastfeeding and complementary feeding practices for every child is recorded annually. In addition, we may include additional potential confounders in the models based on emerging literature.

### Sample size and Study centers

Assuming an incidence of 5 % obesity in children born to mothers with euglycemia, [48, 49] and a relative risk of 1.5 in the hyperglycaemic group, our estimated sample size for 80 % power to detect a difference at a 95 % confidence level, is 2936. Further assuming a loss to follow-up of up to 60 %, we plan to recruit 5000 women. The assumed loss to follow up is comparable to another birth cohort followed up at a similar age in India (Mysore Parthenon birth cohort); however unlike cohorts recruited from the community, ours is a hospital based study with outcome assessment at routine immunisation visits during infancy which are generally well attended, we expect the eventual loss to follow up to be much lower in our study [50]. We have obtained approval to conduct the study at Jayanagar General Hospital, a 300 bedded hospital. In addition, the city municipal corporation (Brihat Bengaluru Mahanagara Palike : BBMP) governing the greater Bangalore metropolitan area has provided approval for public hospitals in the city to participate in the study. The study is initiated at Jayanagar General Hospital; the hospital registers around 200–400 new cases of pregnant women each month.

### Eligibility criteria

All pregnant women between the age of 18–45 years who are less than 32 weeks of gestation and a) plan to deliver in the study hospital and b) reside in the study area will be eligible for recruitment. The recruitment will be done after obtaining an informed consent and noting relevant socio-demographic details. The exclusion criteria includes history of diabetes, Hepatitis B infection, HIV positivity and inability to complete the oral glucose-tolerance test within 32 weeks of gestation.

### Recruitment and follow-up

**Fig. 3:**
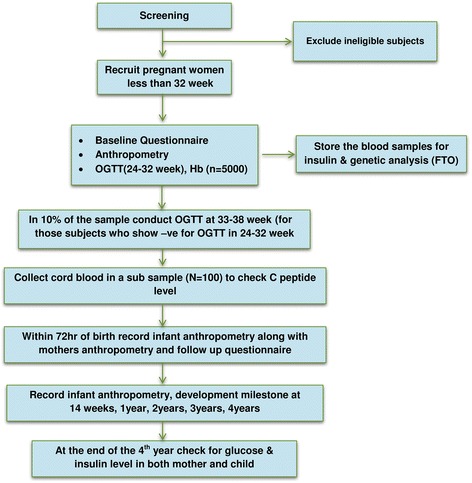
Flow Diagram depicting the steps in cohort study. The whole process of screening of eligible pregnant women for OGTT, recruitment and follow-up at the end of 4^th^ year has depicted in this flow diagram

The process of recruitment, follow-up and corresponding timelines in the cohort have been captured in the Fig. 3. Pregnant women with a gestational period of less than 32 weeks will be recruited. A baseline questionnaire will be administered that includes socio-economic status, Standard of Living Index, 24-hour dietary recall, dietary habits, physical activity, obstetrics history, psychosocial stressors and social support. Weight, height, sitting height, skinfold thickness of biceps, triceps and subscapular skinfold will be recorded (Table 2). Blood pressure will be measured using an automated BP apparatus. All women will undergo an OGTT at 24 to 32 weeks of gestation. They will be asked to fast for a minimum 8 h prior to the study visit, where fasting samples will be drawn. Subsequently, 75 g of glucose will be given and the postprandial sample will be drawn after two hours. In a sub-sample of pregnant women who have normal ranges of glucose in the second trimester, another OGTT at 33 to 38 weeks will be performed to explore the proportion of women who develop gestational diabetes in the later phase of the third trimester. In a sub-sample, HbA1c test will also be conducted based on age, BMI, family history of diabetes and parity. Maternal subcutaneous fat is a significant predictor of adverse pregnancy outcomes. The visceral and subcutaneous fat thickness around abdomen through ultrasound scan will be obtained. A trained sonographer will be performing the scan and strict measures will be taken to avoid gender determination. Visceral fat builds up between and around internal organs such as the stomach and intestines, and produces toxins that make the body resistant to insulin. Subcutaneous fat is found just beneath the skin, and total fat is the combination of visceral and subcutaneous fat. Depending on presence in hospital visits, the husband’s anthropometry and blood pressure will also be recorded.

**Table 2:** Anthropometry and ultrasound measurements in mother and child

Time	Test
24–32 weeks	Blood pressure of mother (Automatic BP apparatus)
24–32 weeks	Anthropometry of mother
	Weight
	Height
	Sitting Height
	Biceps skinfold
	Triceps skinfold
	Subscapular skinfold
	Head circumference
Ultrasound in pregnant women	Subsample of pregnant women (*N* = 500): Visceral and subcutaneous fat thickness around abdomen.
At birth, 14^th^ week and then annually	Anthropometry of child
	Weight
	Length
	Crown rump length
	Head circumference
	Chest circumference
	Waist circumference
	Hip circumference
	Biceps skinfold
	Triceps skinfold
	Subscapular skinfold

The measurements on skinfold thickness and weight for length in infants will be taken at birth and at 14 weeks corresponding to the visit of the mother and child for the purpose of immunization. These measurements will be repeated once in every year for the remaining duration of the study. Follow-up at 1, 2, 3 and 4 years will be on the child’s birthday for all children. At the end of the 4 years of follow up, the glucose and insulin levels in children and mother will be tested (Fig. 3).

### Quality control

For questionnaires and anthropometric measurements, field team will be trained using strict protocols and the accuracy and interobserver and intraobserver reliability of their measurements assessed at the outset and every 6 months. Anthropometric equipment will be calibrated monthly and recordings logged. Biochemical assays carried out by a single laboratory will be quality assured by India’s National Accreditation Board for Testing and Calibration Laboratories (NABL). Quality control samples will be collected for the internal and external quality checks.

### Data collection

Structured questionnaires will be administered by trained interviewers for baseline assessment and follow-up. Data will be entered directly using tablet devices provided to the field staff with validation checks built in to minimise data entry errors. Trained phlebotomists will collect blood samples. Participants not attending follow-ups will be reminded through linkages with frontline health workers in the community.

### Laboratory analysis and sample storage

We will collect 11 ml fasting venous blood sample and 2 ml postprandial sample 2- h after following a 75 g oral load of glucose for the laboratory investigations. The fasting sample will be collected in plain, EDTA and sodium fluoride vacutainers for storage, haemoglobin and glucose assays respectively. Blood samples will be centrifuged and transferred within an hour in cool boxes to a single central laboratory for assays with external quality assurance mechanisms in place. Relevant study assays will be carried out at each time point (Table 3) and remaining sample stored in aliquots for future analysis.

**Table 3:** List of laboratory tests, sample size and proposed biomarkers in the study

Time/Sample	Test	N	Biomarker
Pregnant women between 24–32 weeks	Blood pressure (automated)	5000	
	OGTT (Fasting plasma glucose, 2 Hour Postprandial plasma glucose)	5000	Glucose
	Haemoglobin	5000	Haemoglobin
	HbA1c (glycosylated haemoglobin, Type A1C)	500	Glycosylated haemoglobin
	One aliquot of fasting sample will be frozen at −80 °C for measurement of serum insulin	1000	Insulin resistance and insulin secretion through homeostasis model assessment (HOMA)
	Blood sample to be preserved at−80^0^ C	5000	Micronutrients, DNA and protein markers
	Repeat OGTT after 32 weeks (Fasting plasma glucose, 2 Hour Postprandial plasma glucose)	500	To explore those who develop gestational diabetes after 32 weeks
Cord blood	C-peptide	100	Insulin
Fathers	Fasting blood glucose in fathers	500	Glucose
Mothers (During follow up visits)	Random blood glucose	5000	Glucose
At the end of the study (IV year)	Fasting blood glucose and insulin levels in mother and child	5000	Glucose

### Data management and analysis plan

#### Data analysis

Descriptive analysis will be done to summarize sample characteristics using frequency and percentage. The associations of interest for primary and secondary hypotheses will be assessed using multivariate regression analyses treating skinfold thickness as a continuous outcome as obesity is hard to define at this age, with and without adjustments for potential confounders. For multivariate analyses, variable selection will be based primarily on prior knowledge (a priori) and also the outcome of crude analysis. Variables with *p*-values > 0.10 in the univariate analysis will be included in the multivariate analysis. Possible interactions will also be explored by including product terms in the model [51]. For continuous-variable analyses, odds ratios will be calculated for a 1-SD increase in fasting and 2-h plasma glucose levels. Sum of skin folds >90th percentile will be determined for term deliveries based on gender with adjustment for gestational age and parity (0, 1, and 2+). A detailed plan for data analysis is provided in the Additional file 1.

## Discussion

The findings from this study may help to address important questions on screening and management of high blood sugar in pregnancy. It may provide critical information on the specific determinants driving the underweight-obesity-T2DM epidemic in India, particularly when the data is considered together with data from other cohorts such as the Mysore Parthenon Study, which collected data when India was at an earlier stage of its nutrition transition. In addition it may provide data on appropriate values of cut-offs for healthy glucose levels in pregnancy in India, as well as explorations of role of maternal nutritional status, confounding by psychosocial environment and other determinants.

There can be significant public health implications of the study. First, due to implementation in public health facilities, the results could directly inform the potential impact of scaling up stronger screening and management protocols in the future. Second, the results can position the issues of maternal glycemic control and weight management (both underweight and obesity) to the core of policy agenda. Third, the results may suggest lifestyle modifications in both women and children to prevent and postpone the development of T2DM.

## Additional file

Additional file 1: A detailed plan for data analysis. Conceptual DAG for data analysis and Model-based analysis. (DOCX 133 kb)

## Abbreviations

AC: Abdominal circumference
BBMP: Brihat Bengaluru Mahanagara Palike
BMI: Body Mass Index
BPD: Biparietal diameter
EDTA: Ethylene Diamine Tetra Aceticacid
EPDS: Edinburgh Postnatal Depression Scale
GDM: Gestational diabetes mellitus
GH: Gestational hyperglycaemia
GMHAT: Global Mental Health Assessment Tool
HAPO: Hyperglycemia and adverse pregnancy outcome study
HC: Head circumference
HIV: Human immunodeficiency virus
HOMA: Homeostasis model assessment
IIPH: Indian Institute of Public Health
LMIC: Low- and middle-income countries
MAASTHI: Maternal antecedents of adiposity studying the transgenerational role of hyperglycaemia and insulin
NABL: National Accreditation Board for Testing and Calibration Laboratories
OGTT: Oral glucose tolerance test
SD: Standard Deviation
T2DM: Type 2 diabetes mellitus
WHO: World Health Organization
