# Supplementary material for: Maternal antecedents of adiposity and studying the transgenerational role of hyperglycemia and insulin (MAASTHI): a prospective cohort study: Protocol of birth cohort at Bangalore, India
Source: BMC Pregnancy Childbirth. 2016 Oct 14;16:311. doi: 10.1186/s12884-016-1088-4 (PMC5065083; doi:10.1186/s12884-016-1088-4)
Supplement: Additional file 1: — A detailed plan for data analysis. Conceptual DAG for data analysis and Model-based analysis. (DOCX 133 kb) [file 12884_2016_1088_MOESM1_ESM.docx]

**Appendix.1: Detailed Plan for Data analysis**

For objective 1, we will determine the association between exposure maternal glucose (X) and adiposity in infants (measured by skinfold thickness, Y), and assess the role of the intermediates I (Fetal insulin, birth weight and growth), in mediating this relationship, adjusting for potential confounders Z. These associations are depicted in Figure.1, where the arrows indicate the direction of hypothesized underlying causal relationships.

Z

X I Y

**Figure.4: Conceptual DAG for data analysis of objective.1**

For objective 2, we will determine the association between exposures; psychosocial support (X) and adiposity in infants (measured by skinfold thickness, Y), adjusting for potential confounders Z. These associations are depicted in Figure.2, where the arrows indicate the direction of hypothesized underlying causal relationships.

Z

X Y

**Figure.5: Conceptual DAG for data analysis of objective.1**

**Exploratory and descriptive analyses:** We will estimate frequencies of categorical variables (e.g., Glucose levels, socio economic status (SES), obesity levels etc) over all time points and at birth, 3^rd^ month for time-varying covariates. Similarly, we will summarize continuous variables (e.g., Length for Weight z-score, skin fold thickness etc.) graphically and through summary statistics over all time periods and by time period for time varying covariates. We will repeat these calculations stratifying by education and SES. These descriptive and exploratory analyses will facilitate modeling decisions such as transformation or categorization of continuous measures and suggest the nature of observed changes as a function of the child’s age. Prior to fitting the multivariate models described below, we will estimate marginal associations between exposure (glucose levels, psychosocial support) and outcomes (child adiposity) and between confounders and outcomes.

**Model-based analyses.** With the exception of caste, religion, and a subset of the potential confounders, all variables will be collected multiple times over the course of four years. These serial measurements will allow us to characterize temporal trends in these variables, fine scale temporal dependence of the adiposity measures on the exposure, namely maternal glucose and psychosocial support. We propose to use Bayesian linear mixed models to analyze these data.[1, 2] Given careful choice of the prior distribution, Bayesian methods can overcome the unreliability of likelihood-based variance components estimators.[3] We will employ multiple imputation to handle missing covariate data and will assume that missing adiposity measurements are ignorable.[4]

We will address objective 1 by modeling the relationships between X, I, Y and Z depicted in the graph above via the two component factorization Pr(Y,Z|X,I)=Pr(I|X,Z)Pr(Y|I,X,Z) as described below. We will use the model for I given X and Z to characterize the association between the variables exposure and I X given the confounders. This relationship, depicted as I in the Figure 5, is necessary for I to play the role of mediator. We will use the model for Y given I, X and Z (a) to characterize the direct association between Y and X given Z (by setting the coefficients of I to zero), (b) to determine the strength of the mediating role of I by comparing the adjusted to the unadjusted (for I) coefficients of X, both corrected for Z. For this component, the first level model will be a normal theory regression of an individual’s (indexed by i) quarterly outcomes (Yit) on random effects for his/her quarterly exposures (Xit), mediators (Iit) and time-varying (potential) confounder and fixed effects for the time invariant confounders (Zi). The second level of the model will comprise a set of normal models for the first level random effects parameters.

Specifically,

Level 1: Yit = αi + βi Xit + γiIit + δZi + εit, where εi ~ N(0,Σ) and εi= (εi1, …, εi4) , i.i.d. for subjects i=1, …..4000,

Level 2: αi ~ N(0,a2), βi~ N(0,b2) and γi~ N(0,g2), i.i.d. for subjects i=1, …, 4000.

Where there are several variables of a given class, e.g., mediators, the coefficient, e.g., gi, will be multivariate. The model(s) for I given X and Z will be parameterized similarly, utilizing link functions appropriate to the various I’s.

For objective 2, to control for potential confounding of the association between psychosocial support and adiposity, we will examine a large number of variables simultaneously. For this purpose, we will use multivariate regression analysis. The primary outcome variable is adiposity. The outcome variable(s) being categorical (yes or no), a logistic model is suitable. Variable selection will be based primarily on prior knowledge and also the outcome of crude analysis. Variables with p-value > 0.20 in the univariate analysis were included in the multivariate analysis. Possible interactions were explored by including product terms in the model.

The basic regression model was of the following form:

Y=β_0_ + β_1_X_1_+ β_2_X_2_+. ………+ β_k_X_k_+ 

Where Y represents adiposity, X_i_ are exposure and confounding variables, β_i_ are their coefficients, and ‘ε’ is the random error term. β_0_ is an intercept term that is the average value of adiposity for the entire group that would be detected if none of the exposure variables or confounder variables had any effect. The coefficients indicate the amount of change in the adiposity per unit change in change of the psychosocial support or confounder. We will make conscious efforts to detect collinearity, which occurs when certain predictors are highly correlated with each other. [5]

SAS will automatically exclude subjects with missing values from the regression analysis. This will reduce the effective sample size. Strategies used to handle missing values in regression analysis will depend on the type of missing data. If the data is missing completely at random (MCAR) i.e., the missing cases are independent of the outcome and other covariates; or missing at random [6] i.e., independent of outcome but related to other covariates then we will use the method of multiple imputation to replace the missing values. However, we may use only the subjects with complete data if the number of missing values is very small and seems to be MCAR. In case of nonrandom missing data, we explored the patterns and reasons for the same. Such missing values cannot be simply handled by statistical means. [7]

We will also explore using ordinal logistic regression for all our estimates. We will convert outcomes variables (Y) as ordinals, calculated as the categories ordered in a natural way such as adiposity ordered as absent, overweight and obese. To take account of the ordering obtained from contextual stress domains and data management, we used cumulative probabilities (interrelated to cumulative odds and cumulative logits) model.[8] [9]

For (k+l) ordered categories, these quantities are defined as follows

***P (Y≤i)= p_1_+p_2_+…..+p_i_
odds (Y≤i=i)= [(P(Y≤i) ⁄(1- P(Y≤i)]=[( p_1_+p_2_+…..+p_i_)/(p_i+1_+…+p_k+1)_]***

***logit (Y≤i=i)= ln[(P(Y≤i) ⁄(1- P(Y≤i)], i=1,...,k***

The cumulative logistic model for outcomes arranged in ordinal categories is given by

***Logit (Y≤i=i) = a,+p_i_X.+...+p_im_X_m_, i=l,...,k***

The model is similar to the polytomous logistic regression model except that we have k model equations and one logistic coefficient β_ij_, for each category/covariate combination. The general cumulative logistic regression model therefore contains a large number of parameters. A more parsimonious model can be thought of when the logistic coefficients do not depend on *i* and we have only one common parameter β_ij_ for each covariate. Based on this, the cumulative odds are given by

***Odds (Y≤i=i) = exp(α,) exp****(*β***_i_X****_i_+...+ *β***_m_X****_m_****)* , i=l,...,k**

This model suggests that the k odds for each cut-off category i differ only with regard to the intercepts α_i_. McCullagh coined the term “proportional odds model” [8] as the above model suggests that the odds are proportional. The relatively stringent proportional odds assumption may be especially valid in cases where the ordinal response Y is related to an underlying latent continuous variable.

**References:**

1. Greenland S: **When should epidemiologic regressions use random coefficients?** *Biometrics* 2000, **56**(3):915-921.

2. Greenland S, O'Rourke K: **On the bias produced by quality scores in meta‐analysis, and a hierarchical view of proposed solutions**. *Biostatistics* 2001, **2**(4):463-471.

3. Greenland S, Christensen R: **Data augmentation priors for Bayesian and semi‐Bayes analyses of conditional‐logistic and proportional‐hazards regression**. *Statistics in medicine* 2001, **20**(16):2421-2428.

4. Greenland S, Finkle WD: **A critical look at methods for handling missing covariates in epidemiologic regression analyses**. *American journal of epidemiology* 1995, **142**(12):1255-1264.

5. Afifi AA, Clark V, May S: **Computer-aided multivariate analysis**, vol. 62: CRC Press; 2004.

6. Chandola T, Marmot M, Siegrist J: **Failed reciprocity in close social relationships and health: Findings from the Whitehall II study**. *Journal of psychosomatic research* 2007, **63**(4):403-411.

7. Greenland S, Rothman KJ: **Modern Epidemiology, 3rd edn.** , vol. 2. Philadelphia: Lippincott Williams & Wilkins 2008.

8. McCullagh P: **Regression models for ordinal data**. *Journal of the royal statistical society Series B (Methodological)* 1980:109-142.

9. Altman DG: **Statistics in medical journals: developments in the 1980s**. *Statistics in medicine* 1991, **10**(12):1897-1913.
